# Latest Trends in Lateral Flow Immunoassay (LFIA) Detection Labels and Conjugation Process

**DOI:** 10.3389/fbioe.2022.922772

**Published:** 2022-06-14

**Authors:** Andreea-Cristina Mirica, Dana Stan, Ioana-Cristina Chelcea, Carmen Marinela Mihailescu, Augustin Ofiteru, Lorena-Andreea Bocancia-Mateescu

**Affiliations:** ^1^ R&D Department, DDS Diagnostic, Bucharest, Romania; ^2^ Advanced Polymer Materials Group, University POLITEHNICA of Bucharest, Bucharest, Romania; ^3^ Microsystems in Biomedical and Environmental Applications, National Institute for Research and Development in Microtechnologies, Bucharest, Romania; ^4^ Pharmaceutical Faculty, Titu Maiorescu University, Bucharest, Romania

**Keywords:** LFIA, nanoparticles, bioconjugation, functionalization, diagnostic

## Abstract

LFIA is one of the most successful analytical methods for various target molecules detection. As a recent example, LFIA tests have played an important role in mitigating the effects of the global pandemic with SARS-COV-2, due to their ability to rapidly detect infected individuals and stop further spreading of the virus. For this reason, researchers around the world have done tremendous efforts to improve their sensibility and specificity. The development of LFIA has many sensitive steps, but some of the most important ones are choosing the proper labeling probes, the functionalization method and the conjugation process. There are a series of labeling probes described in the specialized literature, such as gold nanoparticles (GNP), latex particles (LP), magnetic nanoparticles (MNP), quantum dots (QDs) and more recently carbon, silica and europium nanoparticles. The current review aims to present some of the most recent and promising methods for the functionalization of the labeling probes and the conjugation with biomolecules, such as antibodies and antigens. The last chapter is dedicated to a selection of conjugation protocols, applicable to various types of nanoparticles (GNPs, QDs, magnetic nanoparticles, carbon nanoparticles, silica and europium nanoparticles).

## 1 Introduction

Lateral flow immunoassay (LFIA) is a popular, easy to perform and low-cost analytical method which can be used for screening, diagnosis and monitoring of various diseases. For these reasons the applicability of this type of tests is very high, they can be used by any medical staff and even by the patient at home. It is a well-known fact that home-based lateral flow assay devices play a vital role in the management of cardiovascular and infectious diseases. This method gives information regarding the presence/absence or the quantity of a target analyte within minutes from the assay initiation. Although this type of point-of-care (POC) devices were initially developed for the diagnosis of diabetes and to detect the pregnancy hormone, nowadays can accurately detect a series of antigens such as: hormones, vitamins, enzymes, viruses, microorganisms, cardiovascular diseases biomarkers, cancer biomarkers, etc (Andryukov, 2020). They can detect one or more markers/analytes simultaneously.

The principle of LFIA is that liquid, containing the target analyte coupled with the detection label, migrates through the test membrane by capillary force, on which the capture molecules are printed. The interaction between the detection and capture molecules can give a positive or negative result, depending on the presence/absence of the analyte and the test type (sandwich or competitive assay) (Koczula and Gallotta, 2016).

LFIA tests consist of individual segments, namely pads, made of various materials. The sample is placed on a pre-treated sample pad. The dried conjugate is applied to the conjugation pad, which is usually made of fiberglass or polyester, and interacts with the analyte present in the sample, migrating into the reaction zone formed by a porous nitrocellulose membrane (van Amerongen et al., 2018). Depending on the format of the test, the antibody or antigen is conjugated to the detection label. It is then applied to the conjugated membrane and the membrane is dried. The analytical membrane contains immobilized antibodies, proteins or antigens in lines or spots. They serve to capture the analyte and conjugate through specific interactions of the chromatographic process. At the end of the strip is the absorbent pad that collects the excess reagents (O’farrell, 2013).

**FIGURE 1:**
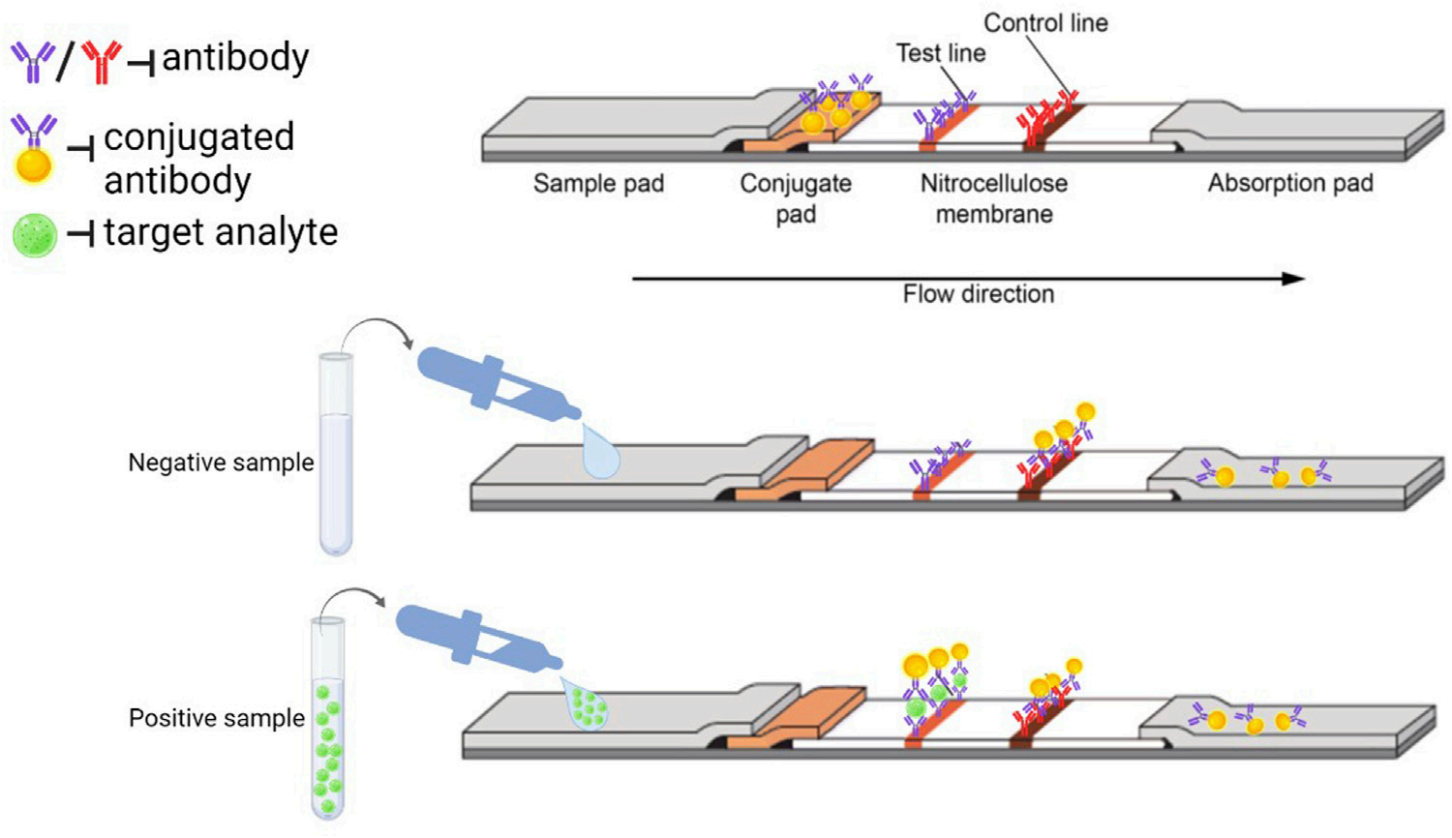
The operation principle of LFIA sandwich-based method (Created with BioRender.com).

Immunochromatographic lateral flow assays are divided into two formats: direct assays (sandwich) or competitive assays (Figure 1). In the sandwich format, the analyte is captured between two complementary antibodies and the presence of a test line indicates a positive result. This type of LFIA is typically used for analytes with multiple antigen sites (hCG, SARS-CoV-2, HIV and others). In the competitive formats, the target analyte blocks the binding sites of the antibody and a positive result is indicated by the absence of a signal in the test line, a negative result when a colored line of any intensity appears on the test line. Competitive assays can be qualitative, semi-quantitative or quantitative and are used for smaller analytes that have a single antigenic determinant (drugs, toxins) (O’farrell, 2013; Andryukov, 2020).

Although the development of an LFIA strip is quite complex and all of the steps, from membrane selection to strip assembly are very important, the efficiency of the conjugation process (binding of the detection label to the detection antibody) is crucial for the device performance. There are a series of detection labels currently used, such as colored particles (gold nanoparticles (GNP), latex microparticles, carbon nanoparticles, luminescent particles (QDs, up-converting phosphor nanoparticles) and magnetic nanoparticles (MNP) (Moyano et al., 2020; Chen X. et al., 2021).

More recently, due to the need to enhance LFIA performance, scientists have reported using amorphous carbon nanoparticles, blue silica nanoparticles and europium nanoparticles with good analytical sensitivity (Zhang L. et al., 2020; Ge et al., 2021; Yin et al., 2022). By using the europium nanoparticles scientists were able to develop a quantitative assay for the determination of Neutrophil gelatinase-associated lipocalin (NGAL), with a detection limit as low as 0.36 ng/ml (Yin et al., 2022). These signal labels can be used to accurately and precisely detected proteins, enzymes, hormones or any other antigen, and also for highlighting the hybridization between nucleic acids. Signal labels must meet a series of requirements such as: excellent stability; efficient and reproductible conjugates, without the loss of the detection molecule activity and functionality; absent or very low non-specific binding and low costs (Koczula and Gallotta, 2016). The conjugation of the signal label to the detection molecule can be done by non-covalent or covalent binding, the last method giving more stable and reproducible conjugates, according to the specialized literature (Razo et al., 2021).

There are a number of articles dealing with signal labels classification, characterization and LFIA applications (Huang et al., 2015; Huang et al., 2019; Di Nardo et al., 2021). The current review is focused on the synthesis and functionalization of LFIA signal labels and offers synthesized working protocols for the conjugation of different particle types, available to both experts in the field and young researchers. We also discuss the influence of the coupling technique in regards of conjugates quality and LFIA performance in terms of sensitivity.

The manuscript is divided into five sections, followed by a discussion section. The first chapter is dedicated to a short introduction in the LFIA concept, structure and functionality with an emphasis on signaling labels. The second part is dedicated to a classification and characterization of the signaling labels and different functionalization methods, including green-synthesis. The last part is dedicated to the conjugation process and protocols, followed by topic related discussions.

## 2 Detection Labels

Detection labels are a substantial component of the overall analytical performance of LFIA in realistic applications. They are used to label antibodies or antigens in LFIA in order to improve their detectability. Bonding stability between the labeling material and the bio component represents a significant factor. Detection labels are stable materials that can go through various functionalization and conjugation strategies in order to improve the affinity parameters of the biocomponent in the detection process of the target analyte.

Over time, multiple labeling materials have evolved in innovative chemistry. These labeling materials include gold nanoparticles (GNPs), latex microparticles (LMPs), magnetic nanoparticles (MNPs), quantum dots (QDs), and carbon nanoparticles (CNPs). In recent studies, scientists have used different detection labels such as silica nanoparticles or europium nanoparticles, to improve LFIA performance.

### 2.1 Gold Nanoparticles (GNPs)

GNPs represent a class of nanomaterials that are widely used in the medical field, in cancer therapy (Ali et al., 2020), diagnostic imaging (Wu et al., 2019), vaccine development (Ferrando et al., 2020), drug delivery (Zhang et al., 2019), food safety (Abedalwafa et al., 2020), biosensing (Hiep et al., 2009), and immunoassays (Fang et al., 2011). GNPs are nanostructures that have excellent physicochemical properties, especially optical properties, and are biocompatible with a wide range of compounds. They were used for the first time in LFIA in the early 1980s (Hsu, 1984; Guo et al., 2020). The detection method provides a macroscopic response based on color observation. The size of the nanoparticles is a critical factor affecting the sensitivity of an LFIA using GNPs, especially influencing the color, respectively the intensity of lines on the strip. The color of gold nanospheres in suspension varies from wine red to dark shades in case aggregation occurs. According to the study by Sahoo and Singh, 2014, the size of nanoparticles depends mainly on the concentration of sodium citrate, but other synthesis parameters such as pH and temperature still have an important role to play. The diameter of GNPs used in LFIA varies from 1 to 100 nm and they come in various forms such as nanospheres, nanoshells (Shabaninezhad and Ramakrishna et al., 2019), nanoflowers (AuNFs) (Zhang et al., 2018), nanocages (Qiu et al., 2020), and nano stars (Xi and Haes, 2019). Gold nanospheres most commonly used by scientists in the optimization processes regarding parameters affecting LFIA sensitivity have diameters in the 20–40 nm range. GNPs with a larger diameter provide a better color observation, but studies show that they are less stable (Wang J. et al., 2019). Kim et al., 2016 investigated the influence of nanoparticles of different sizes obtained by gradually adding, at intervals of 30 min, 1 ml HAuCl4 and 1 ml sodium citrate, in order to better control the size of the nanoparticles. They achieved a higher sensitivity at a nanoparticle diameter of 42.7 ± 0.8 nm for the detection of hepatitis B antigen by LFIA.

GNPs provide rapid detection through LFIA, have a much lower cost compared to reference methods, such as polymerase chain reaction (PCR), and are easy to synthesize. One of the oldest and simplest synthesis methods is the Turkevich-Frens method (Frens, 1973), which can be used to obtain GNPs with diameters in the range of 10–120 nm. The method consists in reducing chloroauric acid (HAuCl_4_) at 100°C with sodium citrate. Nowadays, scientists are looking for green synthesis methods, that can provide better results in a short time, and have low environmental toxicity. Bogireddy et al. (2018) studied the synthesis of GNPs using *Coffea arabica* seed (CAS) extract and chloroauric acid hydrated (HAuCl_4_ x 3H_2_O), at room temperatures, varying the pH of the CAS extract in order to obtain GNPs of different sizes. The chloroauric acid reduction occurred due to hydroxyl groups in the CAS structure. A green method, such as the one studied, is considered a very important discovery in terms of the synthesis of GNPs.

### 2.2 Latex Particles (LPs)

Latex microparticles (LMPs) are spherical particles derived from an amorphous polymer. The most commonly used polymer for their synthesis is polystyrene. A method for synthesizing latex particles of well-defined sizes for various applications is seed emulsion polymerization, which involves dispersion of the monomer in water along with surfactants and a water-soluble polymerization initiator that forms free radicals. Radicals initiate polymerization, the process by which the latex polymer is obtained. This method can be used to achieve dispersion of latex particles with a diameter of less than 0.1 µm (Lovell and El-Aasser, 1997). Another synthesis method, proposed by Eshuis et al. (1991) involves the synthesis of particles by emulsion polymerization, but in the absence of surfactants, in order to eliminate the risk of undesired contamination in the final product. By this method, the scientists obtained particles with a size between 0.5 and 1.2 µm.

Zhu et al. (2019) synthesized, in one step, latex nanocrystals with the surface-functionalized with carboxyl groups by emulsifier-free emulsion polymerization. They used purified styrene, methyl methacrylate (MMA), and sodium methacrylate. The mixture of styrene and MMA was deoxygenated by bubbling with nitrogen, then heated to 70°C in an oil bath in the presence of mechanical agitation. Sodium methacrylate and Vitoria Blue B were added and the mixture was stirred until complete dissolution. Finally, sodium persulfate was added and stirred for 6 h. The obtained microparticles were characterized by scanning electron microscopy (SEM). Spherical, monodisperse particles, with a diameter of 300 nm were observed.

Shen et al. (2021) studied the influence of latex microparticles (LMPs) size and concentration on LFIA performance parameters. The best results were obtained at a diameter of 200 nm, and the line intensity on the strip was most pronounced at an added volume of 25 µl of the suspension of LMPs.

The LMPs surface provides strong physical adsorption for molecular species with hydrophobic regions. They have a uniform surface that allows functionalization with carboxyl or amino groups for the covalent binding of antibodies. The surface is ideal for adsorption of bio components, such as antigens, antibodies, peptides, and oligonucleotides, due to the benzene rings in the styrene structure that predominate on the surface. LMPs are used in various medical applications due to their flexibility and optical properties. In LFIA, they offer the advantage of a wide range of colors, which makes it possible to develop a multiplex LFIA. Kim et al. (2019) developed a two-color multiplex LFIA for the detection of *Plasmodium spp*. differentiated from *Plasmodium falciparum* with an analytical sensitivity of 31.2 ng/ml and 7.8 ng/ml, respectively.

### 2.3 Magnetic Nanoparticles (MNPs)

Magnetic nanoparticles are often used in medical research studies and applications, but also in the geology field or physics. They also have many applications in nanotechnology. They are the active component of ferrofluids for audio speakers, and recording tapes, also the recording media on the hard disk is made up of MNPs assemblies (Kodama, 1999).

MNPs have an advantage over other detection labels in LFIA. Due to their paramagnetic properties, the LFIA detection process can be controlled. In their study, Ren et al., (2016) used magnetic probes for labeling and an external magnet placed under the strip. They relied on the concept of magnetic focusing and used the external magnet to control the movement, respectively the interactions between the elements on the strip. The results obtained were surprising. The externally generated magnetic field enhanced the analytical performance parameters of the classical LFIA. The researchers achieved an analytical sensitivity of ∼23 CFU/ml for *E. coli* O157: H7 and ∼17 CFU/ml for *Salmonella typhimurium*, while the reaction time increased up to 30 min, compared to the common 15 min reaction time (Ren et al., 2016).

In general, particles of magnetite (Fe_3_O_4_) and maghemite (γ-Fe_2_O_3_) are used in medical applications. The most commonly used synthesis method is co-precipitation. Iron oxides, such as Fe_3_O_4_ and γ-Fe_2_O_3_, can be obtained from aqueous Fe^2+^/Fe^3+^ salt solutions by adding a base solution in an inert atmosphere at room temperature or at high temperatures (Abu-Dief and Abdel-Fatah, 2018). Another important synthesis method is hydrothermal synthesis. This technique involves a reaction between a solid and a liquid in a solution but requires very high boiling temperatures and vapor pressure (Abu-Dief and Hamdan, 2016). Green synthesis methods are used by scientists in order to minimize the high toxicity of chemical synthesis. The method proposed by Karade et al. (2018) involves the reduction of ferric nitrate solution with green tea extract and ethylene glycol, which is used as a reducing agent and also as a solvent. Particles with a diameter of 20–25 nm were obtained and have been characterized by X-ray diffraction (XRD) and field emission scanning electron microscope (FESEM). It was observed that as the reaction time increases, the size of the nanoparticles formed also increases.

### 2.4 Quantum Dots (QDs)

Quantum dots are fluorescent label materials. Compared to conventional fluorophores, QDs have unique properties such as photostability, stronger fluorescence, and a more flexible surface in terms of their modification (Savin et al., 2017). QDs have been found to emit 20 times more light than conventional fluorophores and are 100 times more stable (Michalet et al., 2005).

QDs have attracted the attention of scientists in recent years due to their constant and unique optical properties. In general, quantum dots are composed of group III-V and group II-VI elements. The most commonly used materials for the synthesis of these particles are cadmium selenide (CdSe), indium arsenide (InAs), and cadmium telluride (CdTe) (Tang et al., 2021). Despite their unique properties, cadmium-based quantum dots exhibit increased toxicity, similar to GNPs (Li et al., 2020).

Surana et al. (2014) used a more environmentally friendly synthetic method to prepare cadmium selenide quantum dots. A solution of sodium selenosulfate (Na_2_SeSO_3_) was prepared and subsequently injected into a solution of cadmium acetate (Cd (CH_3_COO)_2_) heated at 30°C. Post-injection, the solution color changed from milky white to lemon green. Finally, a solution of 2-mercaptoethanol was added, for strengthening stability, and stirred for 10 min. The process was repeated for different temperature values. The obtained QDs were characterized by UV-VIS absorption spectroscopy, photoluminescence spectroscopy (PL), transmission electron microscopy (TEM), and X-ray diffraction (XRD) (Surana et al., 2014).

Ginterseder et al. (2020) obtained indium arsenide quantum dots by a synthetic method that does not require pyrophoric precursors. They used a simple hot injection method with the main precursor iodine monochloride (In(I)Cl), which also serves as a reducing agent in the synthesis. It reacts with a tris(amino)arsenic precursor, and finally, indium arsenide is produced.

For the synthesis of cadmium telluride quantum dots, Alvand et al. (2019) proposed a green synthesis method using *Ficus Johannis* fruit extract. They used two extraction methods: microwave-assisted extraction (MWAE) and ultrasonic-assisted extraction (UAE). The extract was later used as a stabilizer in the synthesis. The pH of the obtained *Ficus Johannis* fruit extract solution was adjusted to 9. A hydrated cadmium nitrate solution (Cd (NO_3_)_2_ × 7H_2_O) was added under constant stirring at room temperature and under a nitrogen atmosphere. Then, a tellurium solution (Te) was added in the presence of sodium borohydride (NaBH4). The obtained results show that the MWAE is an efficient, fast, and environmentally friendly technique for the synthesis of CdTe QDs, and is also suitable for the synthesis of other nanoparticles.

### 2.5 Carbon Nanoparticles (CNPs)

Carbon nanoparticles are nano-sized carbon elements. They represent a class of nanomaterials that have recently been used in applications in the medical field as carriers for drugs, in imaging or biosensors, and also in the engineering field (Ray and Jana, 2017) or agriculture (Shojaei et al., 2019). CNPs are stable nanoparticles and have a high specific surface area, being considered an ideal candidate for labeling biocomponents (Guo et al., 2020). Although they have a high specific surface area, the number of functional groups which these nanoparticles can be coated with is quite low, making the covalent binding of bio components, such as proteins or nucleic acid sequences, practically impossible. These can be linked to the functional groups on the CNPs surface by physical adsorption, with the advantage that specificity is maintained.

Wang Z. et al. (2019) developed a sandwich format LFIA with CNPs for the detection of *Salmonella enteritidis*. The analysis was appreciated by researchers, who achieved a detection limit of 10^2^ CFU/ml, by a much simpler and less expensive method than the classical sandwich format LFIA.

One method for synthesizing CNPs is laser ablation of graphite. In this process, a piece of carbon graphite is heated to a high temperature where the carbon atoms split off occurs, and they reassemble on a cooled surface in the form of nanoparticles. Similarly, CNPs are formed by chemical vapor deposition (CVD), but instead of the graphite piece, hydrocarbon gas is used as the source of carbon atoms, which separate either thermally or in the presence of plasma (Shojaei et al., 2019).

Ghosh et al. (2021) synthesized fluorescent carbon nanoparticles by controlled carbonization of biomolecule-based carbon precursors with a mixture of ethylene glycol and sodium phosphate. The obtained nanoparticles were purified by acetone-cyclohexane-based precipitation/redispersion method and then functionalized with PEG-diamine or arginine for further bio applications.

### 2.6 Silica Nanoparticles (SiNPs)

SiNPs are silicon dioxide nanoparticles, achievable in an amorphous or crystalline state, with a spherical shape, that can be used in both nonporous and mesoporous forms in nanomedicine applications (Karomah, 2021). Mesoporous SiNPs have attracted the attention of scientists due to their special characteristics, such as a large surface area with variable sizes pores (Wang et al., 2015). SiNPs are highly biocompatible, have a surface that can be easily functionalized, and have a high chemical reactivity that facilitates the staining of these particles. Due to these properties, they have the potential to become a detection label with applications in LFIA. Lirui Ge et al. (2021) reported the first use of silica nanoparticles in LFIA, achieving a detection limit of 10–5 (0.01 IU/ml) for *Brucella* spp. antibody detection.

Through controlled synthesis processes and various functionalization methods, researchers have succeeded in improving the biocompatibility and stability of SiNPs (Slowing et al., 2007). Thus, they have become nanomaterials capable of controlled drug delivery and detection of target analytes in biomedical applications, such as LFIA. Gao et al. (2021) used in their study dendritic mesoporous SiNPs to enrich QDs in order to improve the analytical sensitivity of LFIA. Following optimization, they obtained an analytical sensitivity of 10 pg/ml for CRP detection.

One of the most common synthesis methods for SiNPs is the Stöber method (Stöber et al., 1968), which involves the condensation of tetraethyl orthosilicate (TEOS) in ethanol, under alkaline conditions at room temperature. Ammonia is used as a catalyst, contributing to the formation of spherical-shaped particles. Rossi et al. (2005) developed luminescent SiNPs with applications in bioanalysis, by the Stöber method in the presence of a fluorophore. The synthesis had an 80% yield.

Yadav and Fulekar (2019) proposed a green synthesis method from silico-aluminous class F fly ash. It is a two-step synthesis method compared to the one-step Stöber synthesis. In the first part of the synthesis, silica was extracted from fly ash in form of sodium silicate after sodium hydroxide treatment. In the second part of the synthesis, SiNPs were obtained by the alkali dissolution method. After purification, SiNPs were characterized by various methods and the results revealed highly purified nanoparticles were obtained (90–96%).

### 2.7 Europium Nanoparticles (EuNPs)

Europium oxide (Eu_2_O_3_) nanoparticles have the appearance of a white powder and cause irritation to the eyes, skin, and respiratory tract. They are thermally stable particles, with a melting temperature of 2,350°C, insoluble in water, and partially soluble in strong mineral acids (Marcu et al., 1981). Europium (III) is the most widely used lanthanide for the synthesis of luminescent nanoparticles used in immunoassay and imaging applications (Syamchand and Sony, 2015). Using EuNPs, Wang et al. (2021) developed an LFIA for the simultaneous detection of three different types of antibiotic residues of veterinary drugs: tetracyclines, sulfonamides, and fluoroquinolones, which are common food contaminants. The analysis allowed both qualitative and quantitative detection. For qualitative analysis, the following detection limits were determined: 3.2 ng/ml for tetracyclines, 2.4 ng/ml for sulfonamides, and 4.0 ng/ml for fluoroquinolones. Quantitatively, the EuNPs fluorescence intensity was scanned in the regions of detection lines.

Wakefield et al. (1999) synthesized europium nanoparticles with less than 50 nm diameters by a colloidal precipitation method. A solution of trioctylphosphine oxide (TOPO) was added to a methanolic solution with europium chloride hexahydrate (EuCl_3_ x 6H_2_O), to form a surface layer, in order to eliminate recombination effects at the particle surface and control the size of the nanoparticles. After 10 min of stirring, the nanocrystals precipitated after the addition of a methanolic sodium hydroxide solution. In her study, Kweyama (2018) proposed a green synthesis method using *Hibiscus sabdariffa* extract to obtain EuNPs. The synthesis was carried out in two steps. First, a *Hibiscus sabdariffa* extract was prepared. Then, the extract was used to chelate europium (III) nitrate pentahydrate. The obtained precipitate was dried at 100°C. The results achieved after the characterizations were satisfactory, but the analysis by high-resolution transmission electron microscope (HRTEM) showed that the obtained nanoparticles were not well dispersed.

### 2.8 Up Conversion Nanoparticles (UCNPs)

These nanoparticles can be obtained through a process called up conversion. Up conversion is an optical process based on an anti-Stokes process in which two or more low-energy photons are sequentially absorbed by up conversion materials, followed by the emission of a single photon of a shorter wavelength (Wilhelm, 2017). This process has many advantages, including low toxicity, high chemical stability, large anti-Stokes shift, and low light scattering (Zhang and Han, 2016). Upconverting nanoparticles are a class of luminescent nanomaterials doped with lanthanide ions. They are excited by light in the near-infrared range (usually 800–1,000 nm) and can be modified to emit in the infrared, visible, and UV ranges, depending on the absorption requirements of the photosensitive component (Andresen et al., 2019). The synthesis of up conversion nanoparticles is directly related to the synthetic parameters of the methods, such as reaction temperature and time, pH, and concentration of precursors and surfactants. Modification of these parameters can result in some control over size, morphology, phase, composition, and size distribution. In recent years, a number of synthesis methods have been developed, including coprecipitation, thermal decomposition, hydrothermal and solvothermal methods (Tao and Sun, 2020). Thermal decomposition is a method in which organometallic precursors (rare-earth-based organic salts) are decomposed in heated organic solvents in the presence of surfactants. The principle of the method is that the C-F bond of the organometallic precursor breaks when the reaction temperature is suitably high. The most commonly used organometallic precursor is trifluoroacetate, and the most commonly used organic solvent is octadecene (ODE). Surfactants used include oleic acid (OA), oleyl amine (OM), trioctylphosphine oxide (TOPO), and oleate salts (Tao and Sun, 2020).

Hydrothermal/solvothermal synthesis uses rare earths (water, fluoride, or organic solvent) and a capping ligand to form a homogeneous solution. Rare earths include rare-earth-based oxides, nitrates, chlorides, and acetylacetonates. Fluoride precursors are usually HF, NH4F, NaF, and KF, while EDTA is usually used as a capping ligand. The method is based on a phase transfer and separation mechanism. The metal ions transfer from the liquid solution to the solid phase and react with anions to form nanoparticles. [4] In coprecipitation synthesis, inorganic salts are used to generate positive and negative ions. Surfactants are also added to control nanoparticle growth and prevent aggregation. This method can be used to obtain rare Earth cations from rare-earth-based salts and fluorine anions from NaF and NH4F. Coprecipitation can also be carried out in an organic solvent, obtaining rare Earth cations from oleates, acetates, chlorides, and nitrates based on rare earths (Tao and Sun, 2020).

Upconverting nanoparticles have been used as reporters on rapid lateral flow immunoassay in a few applications. Martiskainen et al. (2021) [5] developed a rapid test for the diagnosis of hepatitis B virus surface antigen, using upconverting nanoparticles as reporter. The assay reported improved specifications than the conventional visually read LFIA, the limit of detection being 0.1 IU HBsAg/ml in spiked serum, compared to 3.2 IU HBsAg/ml. In terms of sensitivity, the conventional LFIA had 87.7% (95%CI: 79.9–93.3%), while UCNP-LFIA showed (95% CI: 89.5–98.5%). Bayoumy et al. [6] propose a quantitative test for cardiac troponin I using UCNP-LFIA. The performance of the developed assay was evaluated using plasma samples and compared the results with two reference tests. UCNP-LFIA’s limit of detection was 30 ng/L and limit of blank 8.4 ng/L.

### 2.9 Green Synthesis

Green synthesis methods have been listed for each class of nanoparticles. The role of these synthesis methods is to eliminate the risks of using chemical reagents that are potentially hazardous to the environment. Green synthesis methods involve the use of non-toxic reagents, consume less energy in the manufacturing process and the resulting compounds are more environmentally friendly. For these reasons, green synthesis has attracted the attention of researchers in recent years. Although they offer a wide range of advantages for the synthesis itself, extensive studies are needed in order to become a candidate with greater potential than nanoparticles obtained by classical methods. In the case of nanoparticles synthesized by green methods, various problems (Ying et al., 2022) are reported such as their lower stability and the fact that chemical reagents with increased toxicity cannot be completely removed for some particles synthesis. Reaction conditions are also more difficult to control, with many particles necessitating purification steps and making it very difficult in some cases to separate secondary compounds from the reaction medium.

There is still insufficient data on the impact of the use of green synthesized nanoparticles on LFIA performance parameters, however, gold nanoparticles and cadmium free QDs obtained by this method revealed some good characteristics, suitable for this kind of application. GNPs obtained by green synthesis were used for the development of a LFIA for the detection of *Listeria* monocytogenes, with a detection limit of 2.5 × 10^5^ CFU/ml for pure culture and 2.85 × 10^5^ CFU/ml in pork tenderloin sample. The obtained nanoparticles were spherical, had 20–28 nm and showed higher salt stability than nanoparticles obtained by classical synthesis (Du et al., 2020). Cadmium free QDs structures had low toxicity, good stability and photostability and were used to develop a rapid test for the detection of C-reactive protein (CRP), with a 5.8 ng/ml detection limit (Wu et al., 2018). In conclusion, there are a series of methods for the synthesis of labeling probes, however their suitability is to be determined according to the application and although each have their advantages, they also have some drawbacks. The advantages and drawbacks of the synthesis methods for each particle type are presented in Table 1.

**TABLE 1:** Pros and cons of the synthesis methods for each type of detection label.

Detection label	Synthesis method	Advantages	Disadvantages
GNPs	Turkevich-Frens method	- simple and reproducible technique	- GNPs with a diameter greater than 30 nm lose their spherical shape; Dong et al. (2020)
		- stable GNPs with controlled size are obtained	- at suboptimal reagent concentrations, pH, or temperatures, GNPs lose their stability
		- the method is applicable to a wide range of precursors	
	Green synthesis	- more environmentally friendly	- involves several steps in the synthesis in general, as it adds the step of extracting the active compound from the CAS
		- it is a rapid and low-cost method	- it is difficult to determine which are the reactive compounds in the extract
		- reaction parameters are much easier to control	
LMPs	Seed emulsion polymerization	- products are obtained in latex form that is ready for use	- if excessive amounts of seeds are added, bimodal latexes with low viscosity can form
		- the latex particles obtained are more stable	- surfactants and other additives remain on the particle surface and are difficult to remove
			- organic compounds increase toxicity
	Emulsifier-free emulsion polymerization	- the absence of an emulsifier eliminates the risk of undesired contamination in the particles obtained	- larger diameter particles are obtained due to the hydration layer on the surface
		- synthesis involves a single step	
		- spherical, monodisperse microparticles are obtained	
MNPs	Co-precipitation	- is an efficient method	- washing, drying, and calcining cycles are required to obtain a pure compound
		- the process can be easily controlled	- pH adjustment may be necessary
		- particles of well-defined size and properties are obtained	
	Hydrothermal synthesis	- a process that occurs with excellent control over the size and shape of nanoparticles	- requires high temperature and vapor pressure
		- involves minimal waste	- require expensive equipment and installations
	Green synthesis	- the reducing agent is a natural compound	- to obtain larger particle diameters, the reaction time increases
		- exhibits low toxicity	
QDs	CdSe synthesis	- synthesis occurs at room temperature	- increased cadmium toxicity
		- stability is improved	
	InAs synthesis	- pyrophoric precursors are not required	- some of the compounds involved are highly toxic
		- In(I)Cl is used both as a reducing agent and as a source of indium	
	CdTe synthesis—green synthesis	- the extraction method of the reactive natural compound has been shown to be useful for other nanoparticle syntheses	- requires nitrogen atmosphere
		- it is a fast and efficient method	- cadmium is highly toxic
CNPs	Laser ablation of graphite	- nanoparticle size can be controlled	- use of the equipment requires qualified operators
		- good dispersity is achieved	- the cost of the equipment is high
		- high efficiency	
	Chemical vapor deposition	- high purity particles are achieved	- secondary reaction products are highly toxic
		- low pressure is required	- nanoparticle deposition is achieved at high temperatures
	Carbonization	- the process can be easily controlled	- requires nanoparticle purification
		- biological precursors are used	- sodium phosphate has negative effects on the human organism
SiNPs	Stöber method	- occurs at room temperature	- ammonia, used as a catalyst, is highly toxic
		- monodisperse nanoparticles are obtained	
	Green synthesis	- the final compound obtained reaches a purity of 90–96%	- method involves two steps
		- exhibits low toxicity	- purification of the nanoparticles is required
EuNPs	Colloidal precipitation method	- is a versatile method	- reaction waste products are a negative factor for the environment
		- the method requires mild synthesis conditions	- the method is limited regarding the size of the nanoparticles obtained
	Green synthesis	- more environmentally friendly	- nanoparticles obtained are not well dispersed
		- crystalline nanoparticles are produced	- requires purification steps

## 3 Functionalization Methods

In order to bind certain ligand molecules, specific for the target analyte, signal molecules must be functionalized with compounds carrying functional groups such as -COOH and -NH_2_, because covalent binding generates much more stable conjugates than physical absorption. Various methods have been reported, and their applicability depends on the signal molecule and/or ligand type.

### 3.1 Gold Nanoparticles

Ligands with thiol, amine or carboxyl groups can be used to increase GNPs solubility and bind different proteins or antibodies. One study reported using 1-dodecanethiol for the functionalization of GNPs immediately after reduction. The binding was fast and the nanoparticles precipitated from the diglyme solution. The particles appeared to be dispersible in a series of nonpolar solvents. In order to make them water soluble, they used 2- (dimethyl amino) ethanethiol hydrochloride, added to a dichloromethane solution. They concluded that amine ligands generated larger particles than the thiol ligands (Schulz-Dobrick et al., 2005). GNPs can also be modified using amino acids such as cysteine. Citrate stabilized GNPs can form a double layer type structure, in which the cysteine replaces the citrate layer on the gold surface, but aggregation is still a problem (Majzik et al., 2010). Better results were obtained when they covered the particles with Aβ_1-42_ (β-amyloid peptide) and no aggregation was observed (Majzik et al., 2010). Gold particles and nanorods can be effectively functionalized using Poly (ethylene glycol) methyl ether thiol (mPEG thiol). Functionalization with mPEG thiol can easily be performed by adding a concentrated solution of CTAB-AuNR, drop by drop, into a solution of mPEG thiol (5 mg/ml) and leaving it to stir for at least 12 h. The purification can be carried out by tangential flow filtration (TFF) (Lohse et al., 2013).

Another efficient way to introduce functional groups on the surface of GNPs is by immersing the particles in a 11-Mercaptoundecanoic acid (MUA) solution which will generate carboxyl groups, that can be activated using 1-ethyl-3-carbodiimide hydrochloride (EDC) or for a better reaction yield EDC and NHS (N-Hydroxy succinimide) (Fan et al., 2020). However, caution should be taken when establishing the optimum NHS concentration, because excess can lead to aggregation. Citrate stabilized GNPs can also be functionalized using thiol-modified glucose, which has been reported to facilitate the study of the biological recognition of the maltose binding protein (Spampinato et al., 2016). For this purpose, the GNPs were mixed overnight with a 1-ß-D-thio-glucose solution, in order to obtain a self-assembled monolayer on the surface of the nanoparticles, *via* the S-Au bond (Spampinato et al., 2016).

Recent studies show that excellent and robust functionalization of the GNPs can be obtained using calix [4] arenes bearing diazonium groups on the large rim. One study reported that by using a calix [4] arene-tetra-diazonium coupled with four oligo (ethylene glycol) chains, very stable conjugates can be obtained, due to the C-Au bond and also that this method can be an excellent strategy when trying to obtain a well-defined number of functional groups (Valkenier et al., 2017).

Another approach was reported by Harrison et al. (2017), which generated a mix monolayer on the surface of GNPs, using polyethylene glycol (PEG) and one of the following peptides: receptor-mediated endocytosis (RME) peptide; endosomal escape pathway (H5WYG) peptide or the Nrp-1 targeting RGD peptide (CRGDK). The binding of these peptides was mediated by the thiol group of the cysteine residue. They concluded that for each of the tested peptides the optimum pH was around 8.00 (Harrison et al., 2017).

Quinoxaline derivatives, which are an important class of heterocyclic compounds, have also been used for the capping of GNPs. One study showed that GNPs were easily functionalized using 2,3-diethanolminoquinoxaline (DEQX) and 2-(2,3-dihydro- [1,4] oxazino [2,3- b] quinoxaline -4-yl) ethanol (OAQX). They used these conjugates to target cancer cells, because compounds with a quinoxaline motif can bind to phosphatidylinositol-4,5-bisphosphate 3-kinases (PI3Ks) and these enzymes are overexpressed in some types of neoplasms (Araújo et al., 2019). Quinoxaline derivatives have also been used to functionalize silver and gadolinium-based nanoparticles and were shown to give excellent stability, while providing functional groups such as -NH_2_ or -OH, which could serve for the binding of different ligands (Morlieras et al., 2013; Neri et al., 2021).

### 3.2 Latex Nanoparticles

Cationic latex nanoparticles were decorated with amino groups using multistep emulsion polymerization. In the first steps they synthetized monodispersed cationic latex particles and in the last steps, an amino-functionalized monomer (aminoethyl methacrylate hydrochloride) was used to functionalize the nanoparticles. These particles can be used to bind antibodies, after the modification of the amino groups with glutaraldehyde (Ramos et al., 2003). In a recent study the influence of the cationic monomer N-(3-aminopropyl) methacrylamide hydrochloride (APMH) concentration on the polymerization of styrene was evaluated and the results showed that when the concentration of APMH increased from 0.5% to 0.8%, there was a raise in the particles number and a decrease in the particles size, from 600 nm to only 100 nm (Marmey et al., 2020).

Functional groups can be added on latex particles by a reaction called the copper-catalyzed Huisgen reaction, which is representative for the click chemistry concept, that includes reactions that can be performed fast, easily, products are easy to purify and give excellent yields. Based on the Huisgen reaction, polystyrene spheres containing 4-vinylbenzyl chloride were synthetized without any organic solvents, buffers or stabilizers (Breed et al., 2009).

One recent study reported using a new trithiocarbonate holding two alkoxyamine moieties for the controlled reversible addition–fragmentation chain transfer (RAFT) polymerization of acrylic acid. The resulting PAA (polyacrylic acid) was used for the polymerization of styrene and n-butyl acrylate, by polymerization-induced self-assembly (PISA) which resulted in well-defined latex nanoparticles covered with alkoxyamines groups (Thomas et al., 2015).

Polysaccharides coated latex nanoparticles can be an excellent environmentally friendly alternative. As an example, dextran has already been successfully used to functionalize latex nanoparticles. Basically, Dextran end-bearing CTA group was synthetized in two steps: 1) the end chain was functionalized with ethylenediamine (eDexN); 2) eDexN was functionalized using CTA-NHS through an amidation reaction. The performance of the functionalization has been verified by H NMR and the obtained spectrum revealed the absence of peak for the anomeric proton of the terminal glucopyranosyl unit of dextran, that suggested a successful functionalization with ethylenediamine (Castro et al., 2021).

Polystyrene microspheres synthesized using divinylbenzene and ethylene glycol dimethacrylate as cross-linkers were functionalized using glucose by the thiol-ene reaction. These microspheres can used can serve for studying ethylene glycol dimethacrylate or sensing applications (Yang et al., 2013). Size controlled polystyrene particles can be obtained by choosing the optimum synthesis method, for instance studies show that by emulsion and surfactant-free emulsion, particles of 50 nm to 2 µm can be obtained. After the polymerization with styrene and a cross-linker the tert-butyl group can be removed in acidic conditions in order to generate carboxylic acid (Sekerak et al., 2018).

### 3.3 Magnetic Nanoparticles

One of the biggest challenges is the presence of hydrophobic surfactant stabilizer on the magnetic nanoparticles surface (MNP). To this purpose, scientists have tried various surface modifications from the addition of polymers such as PEG, to compounds such as 3,4-dihydroxyhydrocinnamic acid (DHCA) in tetrahydrofuran (THF). By using DHCA the surfactant (oleic acid) was replaced by this compound, which formed and anchor on the surface of the MNP. In the next stage, the MNPs were neutralized with a NaOH solution. The obtained functionalized particles appeared to be very stable over a wide pH range, between 3.00 and 12.00. And of course, that this type of functionalization confers the ability to bind different amine-containing molecules using the carboxyl group (Liu Y. et al., 2014).

Polyethyleneimine (PEI) functionalized MNPs have been reported to bind to different types of bacteria. By studying this interaction, the scientists concluded that Gram-positive bacteria, such as *Staphylococcus aureus* exhibited less capturing ability than a Gram-negative strain such as *Escherichia coli*. The Gram-negative strains cell wall is mostly made of lipopolysaccharides (LPS) and lipoproteins. By partially dissolving LPS using ethanol, scientists observed that the magnetic capturing decreased significantly (Chen et al., 2019).

Human serum albumin (HSA) coated MNPs were obtained by a one-step functionalization process involving diazo transfer followed by *in situ* Cu(I)-catalyzed azide–alkyne cycloaddition (CuAAC). The most important aspect is that the protein, maintained functionality after binding to the MNPs, which suggests that it could be an efficient technique for new and improved diagnostic methods (Polito et al., 2008).

Polymer coated MNPs have also been reported. FeO_4_ magnetic nanoparticles have been functionalized using polypyrrole (PPy), an organic, conductive polymer and this surface modification induced an increase in the saturation magnetization. They concluded that by attaching specific molecular groups to the PPy MNPs, they could serve in a variety of biotechnology applications (Turcu et al., 2008).

Polydopamine-derived magnetic core–shell nanoparticles were functionalized based on their reaction with 4-azidobutylamine. The newly synthesized material was used to link biotin, proline, galactose and dansyl, but can also serve for many other future applications in nanotechnology (Mrówczyński et al., 2013).

### 3.4 Quantum Dots

Quantum dots (QDs) have some important advantages over traditionally used fluorescent dyes, such as a broad excitation spectrum and a narrow emission one, powerful signal, high quantum yield and photostability. Two of the most important challenges when it comes to using QDs in biomedical applications are water solubility and the functionalization. There a series of QDs types based on the material they are made of, such as chalcogenides (selenides, sulfides or tellurides) of metals like zinc, cadmium or lead and more recently carbon (Cotta, 2020). This chapter aims to present some of the latest methods of functionalizing QDs, currently used to obtain bioconjugates with various applications.

#### 3.4.1 CdTe QDs

One study reported using an innovative method of functionalization, with hyperbranched poly (amidoamine)s (HP-EDAMA), which in contrast to previously described methods appears to enhance the photoluminescent properties of the QDs by as much as 2 times. The nanocomposites were prepared using a simple procedure, which involves the preparation of a CdTe solution and adding it to a solution of (HP-EDAMA), drop wise, under vigorous stirring (Shi et al., 2013). CdTe with thioglycolic acid (TGA) and mercapto-acetohydrazide (TGH), synthetized by the hydrothermal method, were functionalized with PEG by forming a hydrazone chain *via* the reaction of aldehyde and hydrazine (Du et al., 2019).

The functionalization of CdTe QDs with 4-mercaptopyridine (4-Mpy) determined an enhanced Raman signal, which is potentially explained by a charge transfer mechanism. These findings suggest that QDs with chemisorption could be used as labels in various biological imaging applications and more. After the preparation of the 4-Mpy functionalized QDs, a portion was concentrated 5 times and 2-propanol was added drop-wise, under stirring, until the solution became turbid, after 20 min, the 4-Mpy QDs were isolated by centrifugation (Wang et al., 2008).

Water soluble CdTe QDs prepared using thioglycolic acid have been modified using ethylene diamine (EDA) and the resulting particles exhibited enhanced fluorescence and photostability and suppressed blinking (Mandal and Tamai, 2011).

CdTe nanoparticles have also been developed for the detection of adenine and guanine, through the functionalization with thioglycolic acid (TGA), but so far only synthetic probes have been used for the validation of these fluorescence probes (Li et al., 2012).

One study focused on developing an optical sensor for the detection of herbicides. In this regard, QDs were functionalized with cysteamine hydrochloride and they found that the fluorescence decreased linearly in the presence of herbicides, according to the Stern–Volmer equation (Mahdavi et al., 2018). Another study designed QDs based probes for the detection of protamine (PT) in drugs and urine samples. The QDs were functionalized with mercaptosuccinic acid (MSA), according to the following procedure: a mixture of cadmium chloride (CdCl_2_) at 100 μmol, sodium citrate dihydrate at 765 μmol, MSA at 100 μmol and sodium tellurite (Na_2_TeO_3_) was prepared; then sodium borohydride (NaBH_4_) (660 μmol) was prepared; 17 ml of ultrapure water was added in a round-bottom flask, afterwhich, the components were added in a precise order; the mixture was heated at 90°C, under reflux; the CdTe-MSA modified QDs were precipitated with ethanol and separated by centrifugation (washed 3 times, 15 min at 3000 RPM). The modified particles were resuspended in 10 ml of water and stored away from light, at 4°C (da Costa et al., 2021).

#### 3.4.2 CdSe QDs

This type of QDs has previously been functionalized using various types of thiol molecules. The most efficient molecules, that also improved their optical properties were 1,4-benzenedimethanethiol (1,4-BDMT), biphenyl-4,4′-dithiol, 1,16-hexadecanedithiol, 1,11-undecanedithiol, 1,8-octanedithiol, and 11-mercapto-1-undecanol (Zhu et al., 2014). Another simple functionalization method for CdSe QDs is through the use of mercaptopropyl acid (MPA) and surface passivation by introducing a ZnS shell. The bioconjugation properties of the MPA functionalized QDs was tested with various antibodies and the results revealed that the obtained conjugates were very stable and the QDs exhibited excellent photoluminescence (Wang et al., 2007).

In order to obtain water-soluble QDs, while not altering the size of the particles too much, hydrophilic molecules can be bound to the hydrophobic ligands on their surface by covalent bonds. Usually, these hydrophilic molecules are carboxylate ligands, because the carboxy group has strong affinity for binding to metals (Yu and Peng, 2002). One group designed a ligand called 5-norbornene-2-nonanoic acid (NB-nonanoic acid), which was introduced during QDs synthesis and the resulting nanocomposite was successfully transferred to an aqueous solution after clicking with tetrazine-PEG. These QDs can be further functionalized by adding different functional groups, depending on the application (Chen et al., 2018).

Ethanolamine-O-sulfate, aminotetraethylene glycol (H_2_N–TEG–OH) and aminotetraethylene glycol azide (H_2_N–TEG–N_3_) were also used for the surface functionalization of CdSe QDs by a one-step procedure. The procedure consists of mixing a QDs solution (7 μM) with each one of the modifiers (concentration 600 mM), the pH was brought to 9.00 with a solution of 2-(N-morpholino) ethanesulfonic (1 M), cooled at 0°C and then about 12.5 μl of a 200 mg/ml EDC solution were added. The mixture was mixed well and incubated overnight at 7°C. Bovine serum albumin was used to demonstrate the efficiency of the modified QDs in the conjugation process (Ranishenka et al., 2020).

#### 3.4.3 (Cu:InP) QDs

Recently, copper ion-doped indium phosphate (Cu:InP) QDs have been synthetized and functionalized *via* ligand exchange with dihydrolipoic acid (DHLA) and dihydrolipoic acid-polyethylene glycol (DHLA-PEG), which resulted in biocompatible and water-soluble QDs, with high brightness, photostability and a slight increase in the particle size from 2.1 ± 0.5 nm to 2.6 ± 0.2 nm (Xu et al., 2019). The conjugation properties of DHLA functionalized Cu:InP QDs has previously been evaluated by binding biotin on the surface after activation of carboxyl groups with EDC and S-NHS and coupling them with streptavidin-agarose beads. The transmission electron microscopy (TEM) and X-ray diffraction (XRD) analysis revealed 3.9 ± 0.4 nm sized particles were obtained and that Cu doping does not affect the structure of the QDs (Lim et al., 2019).

#### 3.4.4 Lead QDs

Lead QDs were first used for the development of gas sensors, such as an NO_2_ detection sensor, which showed a linear response in the range of 0.5–50 and 84 ppm detection limit (Liu H. et al., 2014). The DHLA-PEG ligands were also used for the functionalization of PbS QDs and the results showed that stable and even biocompatible colloidal nanoparticles can be obtained (Zamberlan et al., 2018). More recently, PbS QDs have been covered with cancer marker HER2 specific affibodies and zinc (II) protoporphyrin IX (ZnPP). The modified affibody (Afb2C), by the introduction of two cysteine residues at the carboxyl-terminus, was used as a capping agent to form the Afb2C-PbS QDs. The procedure involved adding Afb2C in 70% (v/v) (NH4)_2_SO_4_, which was pelleted and washed with TRIS buffer and the final pellet was dissolved in lead acetate trihydrate (Pb (CH_3_CO_2_)_2_ 3H_2_O)) solution (0.0167 M). The pH was adjusted to 11.0 and the mixture was stirred for 30 min in N_2_ atmosphere. After this step, a solution of 0.1 M Na_2_S was added and the PbS QDs were obtained, as indicated by the solution color which turned to dark brown (Al-Ani et al., 2021).

#### 3.4.5 Carbon QDs

The functionalization of carbon QDs (CQD) can be easily achieved by the treatment with acid solutions. By refluxing with high nitric acid concentrations for a few hours various oxygen functional groups can be introduced on the surface of the CQD, such as carboxyl, hydroxyl or carbonyl (Dimos, 2016).

Carbon QDs can be functionalized using different nitrogen-containing compounds such as 6-aminohexanoic acid (AHA), 1,6-diaminohexane, N-octylamine, dimethylamine (DMA), and tryptophan. The functionalized QDs were obtained by mixing glucose (2.8 mmol) and each of the above-mentioned reagents (2.8 mmol) in 19 ml of H_2_O and 1 ml of 2 M HCl, under stirring. After mixing for 30 min, the solution was placed in a microwave oven. The black precipitate was resuspended in deionized H_2_O and stirred for 5–10 min, followed by centrifugation (Nallayagari et al., 2021).

The functionalization of CQD with chitosan has also been reported, to serve as a nanoprobe for the detection of trace amount of water in organic solvents. Chitosan gels were used to cover the surface of the QDs after which they were functionalized using 4-(pyridine-2-yl)-3H-pyrrolo [2,3-c] quinoline (PPQ), by covalent binding through the carbodiimide reaction (Pawar et al., 2019).

A new CQD synthetization method, using citric acid as the carbon source and thiourea as N- and S-doping source was developed and the Fourier-transform infrared spectroscopy (FT-IR) characterization of these particles reveled that various functional groups are available on their surface (carboxylic acids, amines, and thiocyanates), which can serve for the covalent binding of different detection molecules (Karingal and Hsu, 2021).

### 3.5 Silica Nanoparticles

The surface of Si QDs has been modified using functional organic molecules, such as N-vinylcarbazole. The functionalization procedure consisted of dissolving N-vinylcarbazole in 15 ml mesitylene, transferring it to a flask coupled with a reflux condenser and then adding 2 ml of Si QDs by injecting it with a syringe. All remaining gases were eliminated and the mixture was subjected to 156°C for 12 h under N_2_. The resulting modified QDs were purified and washed with ethanol to remove excess reagents (Ji et al., 2014).

Silicon nanoparticles have been functionalized with 7-octenyltrichlorosilane (OTS) to obtain oil-soluble particles with vinyl groups, which were subsequently submitted to a mini emulsion polymerization procedure, with styrene, to make them water-soluble. These newly synthetized and functionalized particles exhibited increased photostability and biocompatibility, and could be used as fluorescent labeling samples in a series of biological applications (Mai and Hoang, 2016). Recently, Si nanoparticles have been used for the development of a LFIA test for the detection of human brucellosis. The Si NPs were modified using 3-aminopropyl triethoxysilane (APTES), for the covalent binding of Staphylococcal protein A (SPA), after the activation with glutaraldehyde (Ge et al., 2021). In another recent study, a rapid test for the detection of the prostate specific antigen (PSA) was developed using silver assembled silica nanoparticles. These nanoparticles were functionalized with APTS and NH_4_OH, for the introduction of amine groups and then dispersed in 1-methyl-2-pyrrolidinone (NMP) and the whole mixture was added in N, N-diisopropylethylamine (DIEA). The carboxyl-functionalized nanoparticles were activated with EDC and NHS and added to a NH_2_-PEG600-COOH (1.6 mM) solution. The anti-PSA antibody was subsequently covalently bound to the surface of the modified silver assembled Si QDs, also *via* carbodiimide reaction (Kim et al., 2021).

### 3.6 Europium Nanoparticles

Europium doped silica nano shells (Eu/SiO_2_) were synthetized and their absorption efficiency was increased by the functionalization with poly (ethylenimine) (PEI), in order to increase the positive charge on their surface. This method was employed for the interaction with HeLa cellular line, but can also be applicable to negatively charged molecules such as DNA. The coating procedure consisted in the suspension of the nano shells in 1.5 ml of PEI solution (0.1 mg/ml), followed by stirring for 2 h and collection of the modified Eu/SiO_2_ NPs by centrifugation (Yang et al., 2011).

Europium-quantum dot nano bioconjugates were obtained using cadmium selenide QDs and europium complexes (EuC) and their performance in biotin-streptavidin long-lived photoluminescence applications was assessed. The first step in the preparation of these bioconjugates, is the coupling of EuC and biotin to an amphiphilic polymer such as poly (isobutylene-alt-maleic anhydride) (PMA), followed by the coating of the QDs with the modified polymer. The QD-EuC-biotin complex was found to have excellent properties and potential to become a very sensitive tool for new diagnostic methods and imaging applications (Cywinski et al., 2014).

Europium nanoparticles have been very recently used to develop a LFIA for the detection of neutrophil gelatinase-associated lipocalin (NGAL) in urine samples (Yin et al., 2022). Also, by using europium functionalized carbon dots (CD), a new point-of-care POC device for the detection of dipicolinic acid (DPA), has been developed (Wang et al., 2020). DPA is an anthrax specific biomarker (Li et al., 2017). The synthetized CD contained COOH and NH_2_ groups and were obtained using a previously reported method. Citric acid monohydrate and urea were dissolved in water and the solution was heated for 3–4 min, until the water was evaporated and a dark powder was obtained. The resulting product was purified by chromatography using a mixture of solvents, methanol and methylene chloride, which were later removed under vacuum. The CDs (0.6 mg ml^−1^) were added to a 0.1 M solution of EuCl_3_ and mixed for about 3 h. After dialysis for 2 days, the particles were separated by centrifugation and the resulting supernatant was lyophilized. The resulting probes were resuspended in water at the concentration of 1 mg ml^−1^ (Wang et al., 2020).

### 3.7 Up Conversion Nanoparticles (UCNPs)

A series of functionalization methods have been developed in order to overcome the instability of UCNPs in aqueous solutions and to introduce functional groups on their surface, for the binding of different biomolecules. The main strategies are: ligand modification; layer-by-layer assembly; ligand attraction; and polymerization (Gee and Xu, 2018).

Some of the most efficient and easy methods involve growing a silica shell, which facilitates the functionalization by silanization or the decoration of the UCNPs with gold or silver nanoparticles, followed by the addition of thiol containing molecules (Sedlmeier and Gorris, 2015. Another approach is to use silica coating and then add PEG and obtain the functionalization using hydrothermal treatment. This protocol involves adding ethanol to a UCNP with silica coating solution, 4 ml of deionized water and ammonium hydroxide. This mixture is then stirred for 5 min, whereupon PEG-SilaneMw500 and APTES are added. After stirring overnight, the solution was degassed and purged with N_2_ and heated to 70°C, 100°C and 200°C for 2 days (Wahyuningtyas et al., 2015).

UCNPs were also hydrophilized and functional groups were added to the surface, after direct oxidation of oleic acid ligands, which were converted to azelaic acid ligands (HOOC(CH2)7COOH). This oxidation process has no negative impact on the morphology, composition or luminescence of the nanoparticles (Zhou et al., 2009).

Lanthanide-doped KGdF4 nanocrystals were functionalized using polyethyleneimine and biotin was bound through its carboxyl group to the amine on the particle surface. The prepared KGdF_4_:Ln^3+^ NCs were used to detect trace amounts of avidin, down to a nanomolar scale (Ju et al., 2012).

## 4 Conjugation Techniques

The conjugation methods can be divided into two kinds depending on their reversible/irreversible nature and those are noncovalent and covalent binding. Because each have their advantages and also some flaws, the choice depends on the desired application (Numata et al., 2019).

### 4.1 Non-Covalent Binding

**FIGURE 2:**
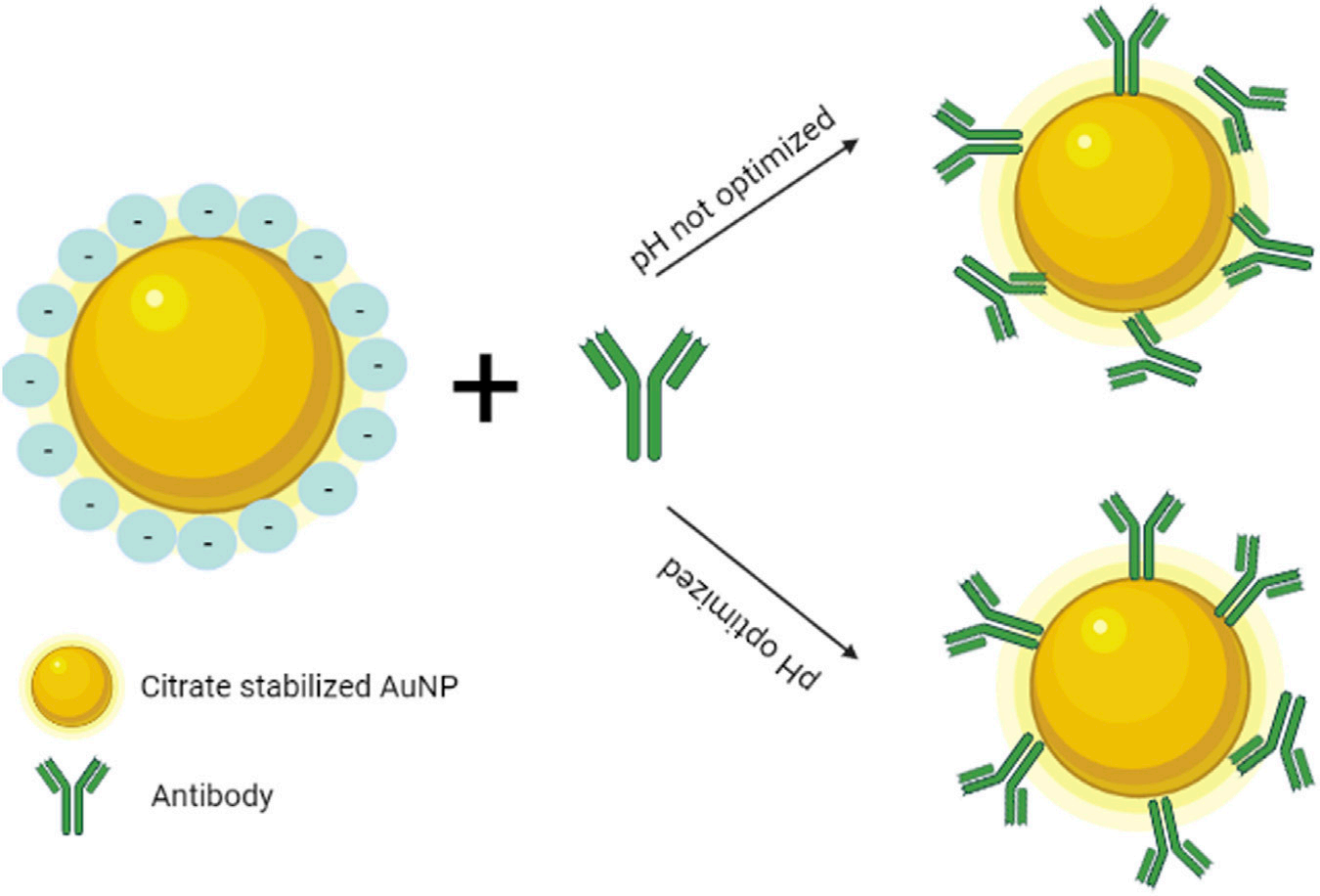
Physical adsorption of antibodies to the surface of citrate stabilized GNP, according to pH (Created with BioRender.com).

For LFIA applications usually the method of choice is physical adsorption, which implies the immobilization of the detection molecules (antibodies, proteins, peptides, etc.) on the surface of noble metals by hydrophobic, electrostatic interactions, hydrogen bonds and van der Waals forces (Razo et al., 2021). Usually, the optimization of this type of conjugation process can be done by testing different pH values near the isoelectric point of the binding molecule (Oliveira et al., 2019). However, conjugation by physical absorption can lead to unreliable results, due to erroneous orientation of the detection molecules (Figure 2), which triggers the blocking of the binding sites and the lack of control on the quantity of absorbed molecules is translated into poor reproducibility (Jazayeri et al., 2016). The orientation of the antibodies can however be controlled, by carefully adjusting the pH between 7.5 and 8.5, which changes the surface charge of the glycoprotein and determines good binding to citrate stabilized GNP (Ruiz et al., 2019). Also, a widely applied method is the biotinylating of antibodies, which will react with streptavidin coated nanoparticles (You et al., 2020).

The optimum quantity of antibody to be added, in order to avoid antigen binding sites blockage or steric hindrance can be determined by the flocculation test, but the final decision should be made after confirming it by testing the selected conjugates on the LFIA strip (Zhang L. et al., 2020).

Reports show that even single domain antibodies, also called nanobodies have been successfully immobilized on GNPs *via* physical adsorption and they found that a pH range of 7.0–9.0 is optimum (Hattori et al., 2012; Goossens et al., 2017).

Physical adsorption was also used for the immobilization of antibodies on nano strings shaped carbon nanoparticles, for the development of a LFIA rapid test for the detection of Influenza A and the results show demonstrated the ability to detect the virus in complex samples, such as allantoic fluid and the cell-associated format (Wiriyachaiporn et al., 2017).

### 4.2 Covalent Binding

**FIGURE 3:**
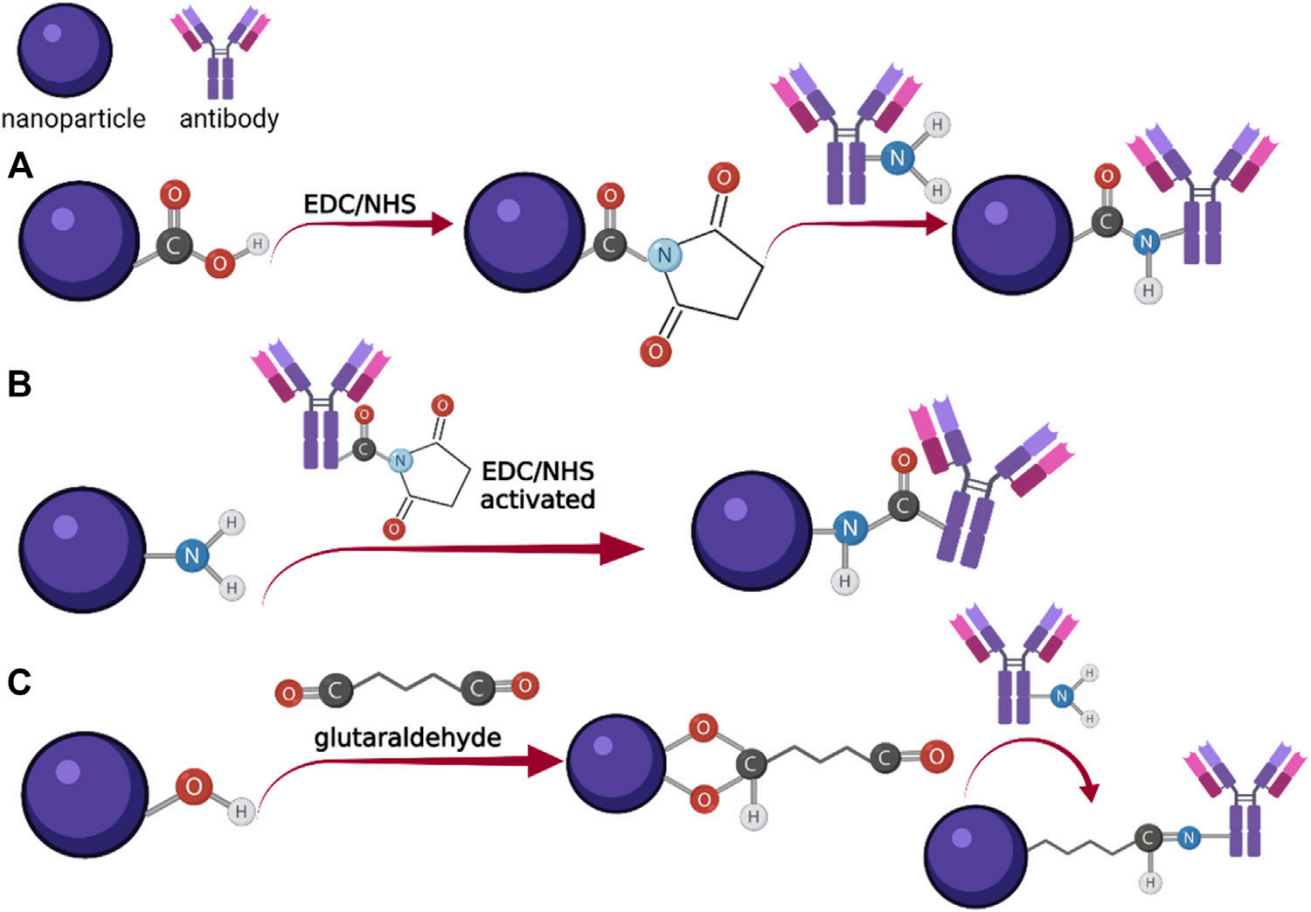
Schematic representation of the conjugation process mediated by the functional groups on the surface of the nanoparticle: **(A)** EDC/NHS mediated coupling of -COOH covered nanoparticle with antibody; **(B)** EDC/NHS mediated coupling of NH2 covered nanoparticle with antibody; **(C)** glutaraldehyde coupling of -OH covered nanoparticle to antibody (Created with BioRender.com).

There are a series of options for the covalent binding of the detection molecules to the labeling probes, but most often various functional groups (-COOH, -NH_2_, -OH) are generated on the surface of the labeling probes by the process of functionalization (Figure 3). Carboxyl or amine covered surfaces can easily be coupled to different proteins through the formation of the amide bond, mediated by EDC and NHS or S-NHS (Oliveira et al., 2019). For the -OH group covered surfaces, the most widely used crosslinking agent is glutaraldehyde, which due to its terminal C-OH groups, can also react with NH_2_ (Kasoju et al., 2018). Oxide surfaces can also be further prepared for the attachment of different biomolecules by covalent bounding with silanes, amines, alkyne and alkene, carboxylates, etc. (Pujari et al., 2014).

**FIGURE 4:**
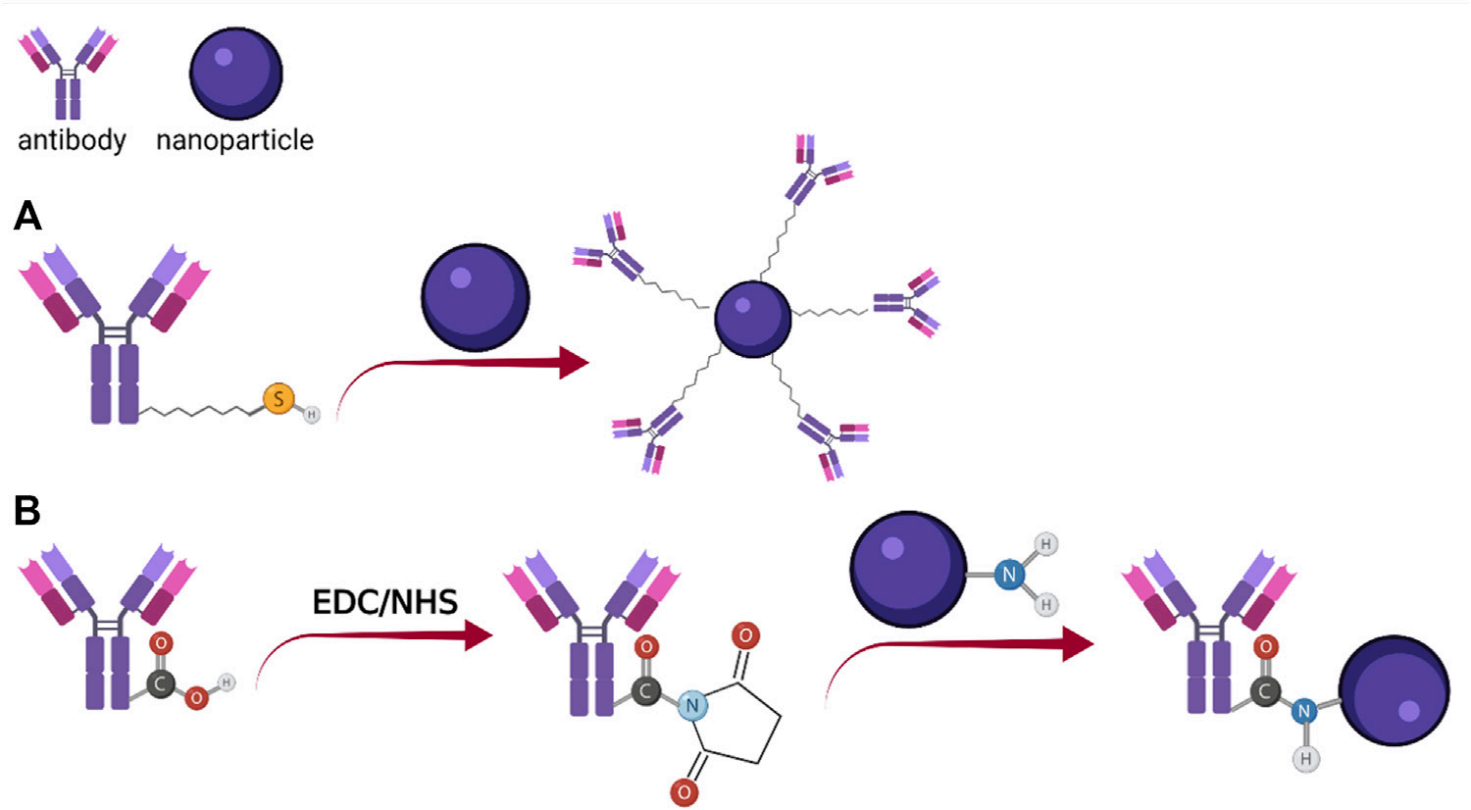
Schematic representation of the conjugation process using chemically modified antibodies: **(A)** Thiol modified antibody coupling with gold nanoparticle; **(B)** EDC/NHS mediated coupling of antibody -COOH with NH2 covered nanoparticle (Created with BioRender.com).

Another approach is to modify the detection molecule (antibody, protein, etc.), by adding a functional group which will react with the surface of the labeling probe (Figure 4). Such an example is the thiolation of proteins and antibodies for the covalent binding on gold surfaces, through the Au-S bond (Sivaram et al., 2017). The Au-S covalent bond is key for obtaining robust and stable conjugates between gold surfaces and thiol modified molecules and for this reason many studies have focused on determining the strength of this interaction and found that it largely depends on the chemical environment (Wei et al., 2015). The generation of sulfhydryl groups from the amino groups, present in the structure of immunoglobulins G (IgG), can be performed using 2-iminothiolane, by a ring-opening reaction (Ben Haddada et al., 2017).

**FIGURE 5:**
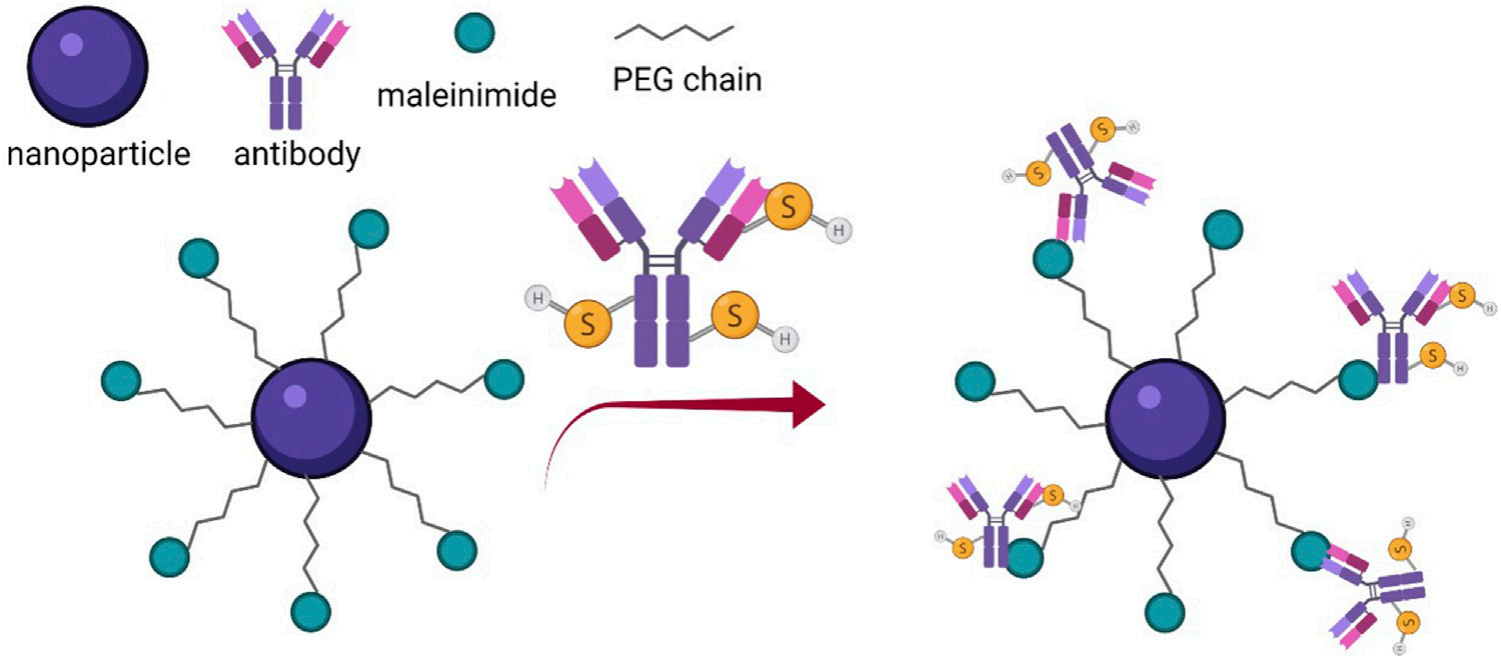
Schematic representation of the conjugation process using chemically modified detection molecule and labeling probe (Created with BioRender.com).

In an attempt to further improve the stability of the conjugates and to obtain highly reproducible results, methods employing both detection molecules and labeling probes modification have been studied (Figure 5). One such example is the coupling of -SH modified antibodies with maleimide functionalized GNP through the Michael reaction (Rayner and Stephenson, 1997). Another innovative method for the covalent binding of antibodies to the surface of GNPs, was developed by modifying the antibody with azido-PEG8-NHS, to generate azido groups and covering the nanoparticles with a thin layer of polydimethylacrylamide (DMA), previously functionalized with an alkyne monomer. The antibody and the GNP were then coupled by Cu(I)-catalyzed azide/alkyne 1,3-dipolar cycloaddition (Finetti et al., 2016).

## 5 Conjugation Protocols

### 5.1 Gold Nanoparticles Conjugation With Antibodies

Parolo et al. (2020) describe the conjugation protocol using 1.5 ml of 20 nm GNPssolution, pH 9 (corrected with 100 mM borate buffer) with the optimized amount of antibody of 2.5 μg/ml. The antibody-GNP mixture was incubated at 650 rpm for 20 min at room temperature using a thermoshaker, then 100 μl of 1 mg/ml BSA solution was added in order to prevent nonspecific absorption for an additional 20 min. After the incubation period, the mixture was centrifuged at 14,000 rpm (30,053 g) for 20 min at 4°C to ensure that unbound antibodies were removed. The antibody-GNP pellet was resuspended in a conjugate pad solution of PBS buffer containing 5% (w/v) sucrose, 1% (w/v) BSA and 0.5% (v/v) Tween-20 and stored at 4°C (Parolo et al., 2020).

To obtain an optimal conjugate, different antibody concentrations are added to the GNPs. If the conjugate aggregates on contact with sodium chloride (NaCl), there is not enough antibody conjugated in the mixture and the color of the GNPs solution changes from red to blue. Panraksa et al. (2020) tested different concentrations of anti-human CRP monoclonal antibody from 0, 10, 50,75, 100, 150, 175–200 μg/ml. Each antibody concentration was homogenized with 200 μl GNPs at pH 8.0 for 30 min, and the yield of the conjugation reaction was tested by adding 80 μl of 10% (w/v) NaCl. After the optimal antibody concentration was reached, 1 ml of GNPs, pH 8.0 was incubated with 100 μl of CRP antibody for 30 min with constant stirring at room temperature, followed by the addition of the blocking solution consisting of 3% (w/v) BSA. The conjugate was centrifuged at 10,000 rpm for 60 min at 4°C, then the supernatant was discarded and 100 μl 3% (w/v) BSA containing 10% (w/v) sucrose was added (Panraksa et al., 2020).

The next GNPs conjugation protocol was obtained by Rodríguez et al., 2016. They developed a protocol to amplify the colloidal gold signal by immobilizing silver salt in a separate pad for a rapid prostate-specific antigen assay. Two types of conjugates were prepared, one consisting of 40 nm GNPs with anti-PSA and the other with 20 nm GNPs and neutravidin. For the first variant, 100 μl 150 μg/ml anti-PSA was added to 1.5 ml GNP solution and mixed for 1 h, then 100 μl biotin-conjugated BSA (40% v/v) was added in the roll of blocking solution. The mixture was centrifuged at 10,000 rpm for 20 min after 20 min of reaction, then the supernatant was discarded and the pellet was resuspended in 100 μl of PBS solution containing 10% sucrose and 1% BSA. The second conjugate was prepared according to the same protocol, with 20 nm GNPs conjugated to neutravidin. The conjugates were stored at 4°C until use (Rodríguez et al., 2016).

A physical adsorption conjugation method was used by Preechakasedkit et al. (2018), in which a hybrid nanocomposite consisting of an GNPs core and a europium (III) chelate fluorophore-doped silica shell (GNPs@SiO_2_ -Eu^3+^) was used as a detection label for human thyroid stimulating hormone (hTSH) assay. First, 1 ml of the nanocomposite suspended in 0.025 M carbonate buffer (pH 9.5) was mixed with 80 µg/ml anti-hTSH antibody for 12 h at 4°C, then 100 µl of a blocking solution containing 0.01 M PBS (pH 7.4), 2% (w/v) BSA and 4% (w/v) sucrose was added and stirred for 6 h at 4°C. The GNPs@SiO_2_ -Eu^3+^-anti-hTSG conjugate was obtained after centrifugation at 8,000 g for 10 min and resuspension of the pellets in 100 µl of 0.01 M PBS (pH 7.4) containing 2% (w/v) BSA, 4% (w/v) sucrose and 0.05% (v/v) Tween 20 (Preechakasedkit et al., 2018).

A new and highly sensitive (LOD 10^−12^ g ml^−1^) LFIA for the detection of sugarcane mosaic viruses was developed using cysteamine modified GNPs. The authors specified that no standard protocols have been reported in regards of cysteamine functionalized gold cationic nanoparticles for use in LFIA applications. Therefore, the protocol developed by them was modified to better suit this application. In the first step, before conjugation, the glycerol was eliminated from the antibody solution, using a 10 kDa centrifugal filter, then 50 µg/ml antibody were added to a 1 ml cysteamine-GNPs solution (OD 1.8). This suspension was mixed for 1 h at room temperature, after which BSA and sucrose were added at concentrations of 0.2% and 2% (w/v) and stirred for a half an hour more (Thangavelu et al., 2022).

### 5.2 Bioconjugation of Quantum Dots With Antibodies

The preparation of conjugates with quantum dots (QDs) is performed in a dark environment to ensure that the QDs do not quench fluorescence in the presence of light.

Foubert et al. (2016) have developed three types of conjugates depending on the functionalization of quantum dots. They hydrophilized the QDs by two methods: with an amphiphilic polymer and with a silica coating. The polymer-coated QDs (250 µl of 2 nmol/ml) were first activated with 25 µl of EDC/sulfo-NHS solution (molar ratio of QD/EDC/sulfo-NHS was 1/200/200) by stirring at room temperature for 3 h. The antibody was added for another 5 h of stirring and incubated overnight at 4°C. Subsequently, different molar ratios of the deoxynivalenol antibody (1/2, 1/5, 1/10, 1/20, and 30) were added per 50 µl of the activated QD solution and centrifuged to separate the unreacted antibody. Carboxyl-functionalized silica coated QDs (carboxyl-QDs@SiO_2_) and epoxy-functionalized silica coated QDs (epoxy-QDs@SiO_2_) were conjugated in the same manner: firstly, activated using EDC/sulfo-NHS solution for 45 min at RT and shaken overnight at 4°C, then different molar ratios of antibody was added to 10 µl of carboxyl-QDs@SiO_2_, respectively 40 µl of epoxy-QDs@SiO_2_ under stirring for 3 h at RT and afterwards stored at 4°C (Foubert et al., 2016).

For the diagnosis and prognosis of lung cancer Chen et al. (2017) have developed a LFIA rapid test based on quantum dot-doped carboxylate functionalized polystyrene nanoparticles, with a detection limit between 0.16 and 0.35 ng/ml. The bioconjugates with the functionalized QDs-doped polystyrene nanoparticles were prepared in two steps. The first consisted of activating 1 ml of QDs suspended in 0.4 ml of MES buffer (pH 6.1) with a solution consisting of 10 µl of EDC solution (10 mg/ml) and 90 µl of sulfo-NHS solution (10 mg/ml). After 30 min of incubation at room temperature, the activated QDs solution was washed 3 times with phosphate buffer (25 mM, pH 7.0) and centrifuged at 8,000× *g* for 5 min. In the second step, the centrifuged quantum dots were resuspended in 0.4 ml of phosphate buffer and 0.1 ml of the antibody solution (1 mg/ml) was added, with gentle stirring for 2 h. Subsequently, 1.3 µl of ethanolamine was added to the reaction mixture for 30 min. The obtained conjugates were stored at 4°C (Chen et al., 2017).

### 5.3 Latex Microparticles (LMPs) Conjugation With Antibodies

The conjugation protocol performed by Raysyan and Schneider (2021) using carboxy latex beads was as follows: In 1 ml of MES buffer (50 mM, pH 6), 100 µl of blue LMP (diameter 0.276–0.325 µm) was added with stirring, followed by the addition of the activation solution containing 24 µl of 200 mM EDC and 240 µl of 200 mM sulfo-NHS, and stirred for 30 min. The obtained solution was centrifuged at 14,000 rpm and 10°C for 7 min and the LMP pellet was resuspended in 1 ml of 50 mM MES buffer (pH 6). In contrast to the conjugation protocol of Shi et al. (2021), the authors sonicated the activated latex particles for 2 min to achieve uniform dispersion. After addition of an optimal amount of antibody stock solution (7 mg/ml in 0.01 M PBS, pH 7.4, 1% BSA, 1% glycerol, 0.02% azide), the solution was incubated for 2.5 h at room temperature. After addition of 30 µl ethanolamine to stop the conjugation reaction, the solution was incubated for another 30 min, then centrifuged at 14,000 rpm and 10°C for 7 min and resuspended in a blocking buffer containing 50 mM Tris with 0.5% BSA (pH 8.0). The LMP-IgG conjugated was used in the development of a LFIA for determination of the endocrine disruptor bisphenol A (Raysyan and Schneider, 2021).

### 5.4 Conjugation of Magnetic Nanoparticles (MNPs) With Antibodies

Smith et al. (2011) synthesized carboxyl-modified iron oxide MNPs, which they conjugated to fluorescently labeled antibodies (Alexa647-chicken IgG) using the following protocol: 250 µl of a stock MNPs solution of 4 mg/ml was washed three times with the same volume of MES buffer at pH 5.0, then 50 µl of a 20-mg/ml EDC solution was added and incubated for 15 min. An amount 100 µg of Alexa647–chicken IgG was added over the activated MNPs solution and incubated for 2 h, vertexing every 15–30 min. The conjugated magnetic nanoparticles were magnetically extracted, washed and concentrated in a 500 µl PBS buffer solution (10 mM) containing 30 mM hydroxylamine and 1% bovine serum albumin and incubated for 30 min. The last step of the conjugation protocol was washing the Alexa647–chicken–MNPs three times with 500 µl aliquots of 10 mM PBS containing 0.05% Tween 20 and 0.1% BSA. Working concentrations ranged from 0.01 to 0.4 mg/ml (Smith et al., 2011).

### 5.5 Conjugation of Carbon Nanoparticles With Antibodies

Polyclonal goat antimouse IgGFcg fragment specific immunoglobulins (GAM) were conjugated on carbon nanoparticles by Suárez-Pantaleón et al. (2013) according to the following conjugation protocol. The first step was sonication of 1% (w/v) carbon nanoparticles prepared in demineralized water, followed by the addition of 100 µl of the GAM solution (175 µg) over 500 µl of a 5-fold dilution of carbon (0.2%, w/v) in 5 mM sodium borate buffer (pH 8.8). The conjugate solution was incubated overnight at 4°C with gentle agitation and then washed four times with 5 mM sodium borate buffer (pH 8.8) containing 1% (w/v) BSA and 0.02% (w/v) NaN_3_ by centrifugation (15 min at 13600 g). The resulting pellets were resuspended in 100 mM sodium borate buffer (pH 8.8) with 1% (w/v) BSA and 0.02% (w/v) NaN_3_. The carbon NPs-GAM conjugate was sonicated for 10 s before use (Suárez-Pantaleón et al., 2013).

The bioconjugation protocol by physical adsorption is simpler and does not require an activation phase of the functional groups. Zhang X. et al. (2020) obtained carbon nanoparticle-based conjugates by sonicating 1 mg of carbon NPs suspended in 1 ml of 0.01 M borate buffer for 10 min, then adding 16 µg of antibody and incubating for 30 min with slow stirring. 40 µl of blocking buffer containing 20% BSA was added under stirring for 30 min, followed by centrifugation at 8,000 *g* for 10 min. The final step was to resuspend the precipitate in 1 ml of 0.01 M PBS solution containing 2% BSA and 20% glycerol and stored at 4°C until use (Zhang X. et al., 2020).

### 5.6 Silica Nanoparticles Conjugation Protocol

Carboxyl-functionalized silver-coated silica nanoparticles (SiO2@Ag@SiO2 NPs) obtained by Kim et al. (2021) were conjugated with mouse monoclonal anti-prostate specific antigen (PSA Ab) by activating the carboxyl group with EDC (2 mg) and sulfo-NHS (2 mg) with stirring for 2 h at room temperature. The mixture was centrifuged and the precipitate dispersed in 50 mM MES. NH_2_-PEG_600_-COOH (1.6 mM) was then added and stirred at 25°C for 2 h. The surface of the dispersed silica-coated silver nanoparticles was blocked by adding 3.2 µl ethanolamine with continuous stirring for 30 min. After the centrifugation at 15,928 rcf for 10 min and redispersion of the precipitate in 50 mM MES buffer, EDC (2 mg) and sulfo-NHS (2 mg) were added and mixed for 30 min. Therefore, SiO2@Ag@SiO2 NP–SNs–PEG-COOH centrifugated and dispersed in MES was added under stirring to anti-PSA Ab and incubated for 2 h at 25°C. Ethanolamine (3.2 µl) was added and stirred for 30 min, then the conjugate was washed several times with 0.5% bovine serum albumin by centrifugation at 15,928 rcf for 10 min and redispersed in 0.5% BSA (Kim et al., 2021).

### 5.7 Conjugation Protocol of Europium NPs

Chen K. et al. (2021) performed a rapid quantitative assay based on the recombinase polymerase amplification technique in combination with a lateral flow immunoassay for the detection of *Listeria monocytogenes*, *Vibrio parahaemolyticus*, and *Escherichia coli* using carboxylic Europium nanoparticles (EuNPs) as signal molecules. The conjugation protocol of EuNPs with anti-digoxin monoclonal antibody was as follows: activation of 2 mg carboxylic EuNPs dissolved in 800 µl 2-(N-morpholino)-ethanesulfonic acid (0.05 M, pH 8.2) with 30 µl EDC by slow shaking for 30 min, then the solution was centrifuged at 12,000 rpm for 25 min. Subsequently, 1 ml of 10 µg/ml anti-digoxin antibody was incubated at 25°C for 2 h and the conjugate was centrifuged for 2 min at 12,000 rpm. The supernatant was then removed and the collected precipitate was resuspended in 1 ml of preservation solution and kept at 4°C until use (Chen K. et al., 2021).

Another important conjugation protocol of europium nanoparticles is described by Bian et al. (2022). Europium-chelate nanoparticles (EU-CNs) (1 mg) were resuspended in 500 µl solution consisting of 25 mmol/L MES (pH 6.1) and 1.25 mmol/L EDC and 10 mmol/L sulfo-NHS with shaking for 30 min. The activated EU-CNs were centrifuged at 18,000 rpm for 30 min and washed twice with 25 mmol/L Tris, 0.2% Tween-20 (v/v), 0.05% Proclin-300 (v/v) and 0.9% NaCl (w/v) (pH 7.8) solution. The pellets were resuspended in 400 µl 25 mmol/L PBS buffer (pH 7) and the antibody solution (25 μg of antiCys C labeling McAb or RIgG dissolved in 100 μl of binding buffer) was added and mixed for 2 h. The sites left free after the conjugation process were blocked by the addition of 500 μl BSA solution (5%). The conjugate was resuspended in 100 μl of labeling antibody storage buffer (25 mmol/L Tris, 0.9% NaCl (w/v), 1% sucrose (w/v), 1% Trehalose (w/v), 5% BSA (w/v), 0.05% Proclin-300 (v/v) and 0.05% TWEEN-20 (v/v), pH 7.2) (Bian et al., 2022).

### 5.8 Conjugation Protocol for UCNPs

A recently described application involved using UCNPs for the development of an LFIA for the quantitative detection of Troponin I. They used carboxylated UCNPs to covalently bind Mab 625. A 0.5 mg UCNP solution was centrifuged at 20,000 g for half an hour, after that the supernatant was removed and the surface was activated by adding 135 µl of MES buffer, with 2 mM EDC and 30 mM S-NHS. After 15 min, the excess reagents were removed by centrifugation at 20,000 g for 10 min and the pellet was resuspended in 20 mM MES. Subsequently, they were centrifuged again and a solution of 30 ug of antibody and 100 mM NaCl was added. The reaction mixture was incubated with stirring for 30 min at room temperature, and the reaction was quenched by the addition of a 50 mM glycine solution. After a further 30 min incubation step, the nanoparticles were washed by centrifugation twice and a solution of 25 mM borate pH 7.8, 150 mM NaCl, 0.1% NaN3, 2 mM KF, 0.2% BSA was added to the pellet (Bayoumy et al., 2021).

## 6 Discussion

There are various particles and bioconjugation methods used for increasing the analytical performance: a detection limit as low as possible, a better repeatability, reproducibility and stability of the LFIA tests.

Although green-methods are preferred and currently under intense study, still, the classic synthesis methods provide particles with well-defined shapes and sizes, good stability and dispersity. The green synthesis of nanoparticles used in LFIA is more attractive because it does not involve heating, is usually very fast, simple, cost-effective and eco-friendly. However, there is not much data regarding their overall effect on the performance parameters of LFIA. One study developed a rapid qualitative LFIA for the detection of *Listeria* monocytogenes, by using salt-tolerant nanoparticles obtained via green-synthesis mediated by an aqueous extract of Damask rose petals. They obtained a detection limit of 2.5 × 10^5^ CFU/ml for pure suspension and 2.85 × 10^5^ CFU/ml for contaminated pork tenderloin sample (Du et al., 2020).

The easiest and most widely used method for the functionalization of GNPs, involves the use of compounds that contain the -SH group or to which this group has been added through the treatment with thiolation reagents. Some of the methods for the functionalization of latex particles described in this paper are very efficient, however, only a few of them use non-toxic and environmentally friendly compounds such as dextran and glucose. According to the literature, polymers (PEG, PEI, PPy) are the method of choice for the functionalization of magnetic nanoparticles. Some of the compounds used for the functionalization of QDs also enhanced their photoluminescence, for instance: HP-EDAMA, EDA (CdTe); MPA, 1,4-BDMT (CdSe); DHLA, DHLA-PEG (Cu:InP); DHLA-PEG (Pb) and GAM (carbon). Silica nanoparticles can be decorated with amino and carboxyl groups, using APTES and DIEA. Silica nano shells and carbon dots properties have been enhanced after they were functionalized with europium.

The conjugation process is a very sensitive step in the development of an LFIA test, that can affect both reproducibility and sensitivity. Some of the most recent studies have described various conjugation techniques, based on the traditional physical adsorption and covalent binding methods and have obtained acceptable detection limits, for a wide range of nanoparticle types and analytes (Table 2). Although most studies showed that covalent binding is preferred due to increased stability and functionality of the conjugate, some reports revealed that by carefully calibrating the pH according to the isoelectric point of the detection molecule, the binding and orientation of the molecules can be improved.

**TABLE 2:** Binding types and their effect on the conjugate quality and overall sensitivity of LFIA.

Type of interaction	Particle	Effect	Target	LOD	References
Electrostatic interaction	Gold nanoparticle	High rate of bound antibody, but poor orientation	17β-estradiol	500 ng/ml	Oliveira et al. (2019)
Covalent binding	Gold nanoparticle	Lower rate o bound antibody, with good orientation	17β-estradiol	200 ng/ml	
Physical adsorption	Gold nanoparticle	Not available	*Erwinia amylovora*	10^4^ CFU/ml	Razo et al. (2021)
Physical adsorption	Au core—Pt shell nanoparticle	Not available	*Erwinia amylovora*	10^3^ CFU/ml	
Covalent binding	Latex nanoparticle	Not available	*Erwinia amylovora*	10^4^ CFU/ml	
Covalent binding	Magnetic nanoparticle	Not available	*Erwinia amylovora*	10^5^ CFU/ml	
Streptavidin-biotin coupling	Gold nanoparticle	Increased sensitivity for the larger particles (35–50 nm)	*Escherichia coli*	10^1^ CFU/ml	You et al. (2020)
			*Legionella pneumophila*		
Physical adsorption (nanobodies)	Gold nanoparticle	Salt induced aggregation	Not available	Not available	Goossens et al. (2017)
Physical adsorption	Carbon nanoparticle	Stable conjugates	Influenza A	3.5 × 10^2^ TCID_50_ mL^−1^	Wiriyachaiporn et al. (2017)
Covalent binding	Gold nanoparticle	Au-S bound	Staphylococcal enterotoxin A	5 ng/ml	Ben Haddada et al. (2017)
Covalent binding	Gold nanoparticle	Bound between surface alkyne and azido modified proteins	Not available	Not available	Finetti et al. (2016)
Physical adsorption	Carbon nanoparticle	Not available	β–lactams	1–30 ng/ml for most antibiotics and 100 ng/ml cephalexin	Zhang X. et al. (2020)
Covalent binding	Europium III chelate nanoparticle	The binding of the protein did not affect the optical properties	Cystatin C	24,54 ng/ml	Bian et al. (2022)
Covalent binding	Latex microparticles	Not available	Bisphenol A	10 ng/ml	Raysyan and Schneider, (2021)
